# Foreign Psychological Literature

**Published:** 1860-04-01

**Authors:** 


### FOREIGN PSYCHOLOGICAL LITERATURE.

OUR retrospect of Foreign Psychological Literature will embrace the following subjects:—

1. On the Discovery of General Paralysis.
2. On a form of Hypochondriacal Delirium succeeding Dyspepsia.
3. On Sitomania.
4. On the Criminal Responsibility of Hysterical Individuals.
5. On the Mental State in Chorea.
6. On consecutive Cerebral Ramollissement.
7. On Interdiction.

### 1. *On the Discovery of General Paralysis, and on the Doctrines emitted by the first writers upon the Disorder.* By M. BAILLARGER.

TWO orders of facts were successively brought to light before general paralysis was really established.

Esquirol pointed out the frequency of paralytic symptoms amongst the insane. He studied the character of this paralysis, its march and influence on the prognosis. But these symptoms, to which Georget, and, above all, M. Delaye, subsequently paid special attention are, if we adopt Bayle's doctrine, but a part of the disease.

In order to complete its discovery, it was necessary to recognise its initial period, and to show that lesion of the intellect forms as essential a characteristic as paralysis itself. The disease consists in two elements. Esquirol saw but one; Bayle discovered the other. Doubtless the second task was much easier than the first, since attention was thenceforth directed to lunatics presenting symptoms of paralysis; but still, the general paralysis of Esquirol, of Georget, and of M. Delaye, was not the disease which the name now imports, with its double lesion of the intellect and movements having each its proper characters. The merit therefore returns to Bayle of having thus established it in adding two new elements, congestion and grandiose delirium (délire des grandeurs).

No one contests this merit with Bayle; it is not so with Esquirol. Reference has been made to a passage in a work of Dr. Haslam's, and this work is anterior to the first labours of the French physician. Without, however, wishing at all to diminish the credit due to Haslam, I think the objection is not entitled to all the weight which has been attributed to it.

It should be remembered, in fact, that many passages quite as precise have been passed over for ages without remark, and that the one now in question would probably never have been known if the works published in France had not, thirty years after, led to a close research after everything in other books relating to the new disease.

This is not all that is to be said in favour of Esquirol. He not only described the principal symptoms of general paralysis in 1814 and in 1816; but he treated each year in his lectures of paralysis of the insane. This may be gathered from M. Delaye's essay.

But again, at this period Esquirol had learnt to recognise the first signs of the disease, and consequently to predict its development, and the new facts thus passed into practice.

"General paralysis," says M. Calmeil, "is widely prevalent amongst the insane, and it is one of the most fatal complications of vesaniæ. All physicians who make mental disorders their special study have clearly defined opinions on this subject, and each time they are consulted on behalf of a lunatic, they carefully examine whether the pronunciation is free from impediment or accompanied with stammering. They rarely hesitate to pronounce the malady incurable when they are able to verify the existence of paralysis, however slight may be its symptoms."

M. Calmeil wrote this in 1826, and added, "M. Esquirol was the first to direct attention to this point, and he has caused due weight to be attached to the gravity of the prognostic."

"M. Esquirol," says M. Calmeil in another place, "has seen professional brethren of great ability maintain that the tongue was not paralysed when even the pronunciation was so embarrassed that a practised ear could not be mistaken."

Esquirol described in writing the symptoms of paralysis in 1814 and 1816, as Haslam had done before; but that is not his chief merit. He has besides both in his lectures and his practice constantly called attention to this complication, the first and slightest manifestations of which he completely apprehended. To overlook it was thenceforth impossible.

Whenever any discovery in science, great or small, is unrolled, it is rare that we do not find in preceding authors its hidden germs. Far from derogating from the merit of the inventor, this circumstance is a proof of the reality of the facts which he has first brought to light.

Haslam has mentioned the pretentious pride of paralytics, but it was nevertheless Bayle who discovered the relation between ambitious delirium and paralysis, and it was he who gained for it a place in science which no one at the present day contests.

Esquirol discovered general paralysis, because in reality it was he who fixed attention upon the gravity of the prognostic presented in the case of the insane by the first signs of stammering, and because he insisted upon this fact every day both in his lectures and his practice, and taught those about him to verify its importance. Had he done but this, without having written anything in 1814 and 1816 on general paralysis, it would still have been to him that we must have accorded the honour of being the first to establish this order of facts.

In my opinion, then, the names of Esquirol and Bayle should be united, and it is to these two authors jointly that we should attribute the merit of having written this great chapter in the history of mental diseases.

To resume: two very different doctrines have been enunciated by the first authors who wrote upon the subject of general paralysis.

The first is that of Esquirol, of Georget, and of M. Delaye; the second is that of Bayle. In the first it is admitted—

1. That there is room in the nosological system for a new species of paralysis—*Chronic muscular paralysis* (Georget), *Imperfect general paralysis* (Delaye).

2. That this paralysis, like all others, is characterized by but one order of pathognomonic symptoms, the symptoms of paralysis.

3. That this new species of paralysis has this peculiarity, that it is observed almost exclusively as a complication of insanity.

4. That the insane paralytic should always be considered as the subject of two distinct diseases, insanity and general paralysis.

5. That this general paralysis intervenes indifferently in all forms of insanity.

From Bayle's doctrine, on the contrary, we arrive at the following conclusions:—

1. That we must inscribe in the nosological system a new species or form of insanity—*ambitious insanity with paralysis* or *chronic meningitis.*

2. That this new species of madness is characterized by two orders of pathognomonic symptoms:—1. Ambitious delirium under the form of monomania and of mania, and delirium with signs of dementia. 2. A general and progressive paralysis.

3. That insanity and general paralysis observed in the same patient are not two distinct disorders like insanity and scurvy, but two orders of symptoms of one single morbid entity.

4. That general paralysis cannot be considered as a complication of insanity.

5. That the symptoms of general paralysis are not observed in all forms of insanity indifferently, but only in cases of insanity characterized by the predominance of ideas of grandeur and power.

6. Finally, to Esquirol and Bayle belongs the honour of having realized by the discovery of general paralysis the greatest progress that can be pointed out in the history of mental disorders.—(*Annales Médico-Psychologiques*, Jan. 1[illegible].)

2. *On a form of Hypochondriacal Delirium occurring consecutive to Dyspepsia, and characterized by refusal of Food.* By Dr. L. V. Marcé.

Amongst the numerous and varied forms of dyspepsia there are some which should especially attract the attention of psycopathists, on account of the peculiar mental condition thereby determined.

We see, for instance, young girls, who at the period of puberty and after a precocious physical development, become subject to inappetency carried to the utmost limits. Whatever the duration of their abstinence they experience a distaste for food, which the most pressing want is unable to overcome; with others the appetite is not wanting, but the digestion is painful, accompanied with flatulence, lowness of spirits, and discomfort. These two varieties of dyspepsia, which are very common, when they occur in young subjects predisposed to insanity from hereditary antecedents, and rendered still more impressionable by that profound nervous disturbance which accompanies the establishment of the menstrual functions, may, by a process of ideas easy to follow, determine a state of partial delirium. Deeply impressed, whether by the absence of appetite or by the uneasiness caused by digestion, these patients arrive at a delirious conviction that they cannot or ought not to eat. In one word, the gastric nervous disorder becomes cerebro-nervous.

It is easy to foresee the consequences of this new morbid condition. All attempts made to constrain these patients to adopt a sufficient regimen, are opposed with infinite stratagems and unconquerable resistance. The stomach digests perfectly what is committed to it, but in the end it comes to content itself with the feeblest doses of nourishment, until one is surprised that life should survive so long with such slender means of reparation. I have observed several cases (and in these the suspicion of fraud must be altogether discarded) where the patient has lived six months, a year, and even more, upon a few spoonfuls of soup or a few mouthfuls of sweetmeat or pastry daily: in one case observed, the amount of liquid and solid food taken, which was exactly weighed, did not exceed fifty grammes a day. It is true that then attenuation proceeded to the last degree; all trace of adipose tissue had disappeared, and the patients were reduced to skeletons; the teeth blackened, the mouth became dry and the tongue red and furrowed; the constipation was such that it was difficult to provoke the expulsion once a fortnight or once a month of matter hard and ovular; the urinary excretion was almost nil, and the abdominal coat was so contracted as to touch the vertebral column; the skin became dry and wrinkled, the pulse filiform and insensible, and all the symptoms preceding death from inanition were strikingly displayed; the weakness soon became so great that the patients could scarcely walk a few steps without being seized with fainting. The nervous predisposition increases with the debility of the organism; the affective sentiments undergo alteration, and all the intellectual energy centres round the functions of the stomach; incapable of the slightest exertion or of sustaining the least conversation beyond their delirious ideas, these unhappy patients only regain some amount of energy in order to resist attempts at alimentation, and very often the physician beats a retreat before their desperate resistance.

Some of these sufferers, at the end of months or years, and after numerous oscillations in their condition, literally die of hunger. In one case of this description, in which a *post-mortem* examination was made under my eyes, the stomach was perfectly uninjured; the mucous membrane was healthy, without injection or softening; the capacity of the stomach was perfectly normal.

It must not be forgotten, therefore, that by reason of the anatomical integrity of the digestive organs, medical intervention may be most advantageous even when the patients appear devoted to incurability and death. I have seen three young girls thus cured, who were reduced to a most alarming and almost desperate state; it remains then to inquire what are the indications we have to follow, and in what way should medical action be directed.

In reference to the greater number of these cases which have come under my notice, I would venture to say that the first physicians who attended the patients misunderstood the true signification of this obstinate refusal of food: far from seeing in it a delirious idea of a hypochondriacal nature, they occupied themselves solely with the state of the stomach, and prescribed, as a matter of course, bitters, tonics, iron, exercise, hydro-therapeutics, with a view to stimulate the activity of the digestive functions. However apparently excellent these therapeutic measures may be, they always proved insufficient when the malady was in an advanced stage. It is then no longer the stomach that demands attention, for the stomach is well able to digest, and suffers only from want of food; it is the delirious idea which constitutes, henceforth, the point of departure, and in which lies the essence of the malady; the patients are no longer dyspeptics—they are insane.

This hypochondriacal delirium, then, cannot be advantageously encountered so long as the subjects remain in the midst of their own family and their habitual circle: the obstinate resistance which they offer, the sufferings of the stomach, which they enumerate with incessant lamentation, produce too vivid emotion to admit of the physician acting with full liberty and obtaining the necessary moral ascendancy. It is therefore indispensable to change the habitation and surrounding circumstances, and to entrust the patients to the care of strangers. If the refusal of food continues notwithstanding these efforts, it becomes necessary to employ intimidation, and even force. If by this last method a satisfactory result be not obtained, I would not hesitate to recommend the use of the œsophagus sound. But it is necessary to proceed progressively and by degrees. Each day and at each repast the nourishment, be it liquid or solid, should be gradually increased, and it would be even well to weigh the food, in order to proceed with greater sureness and confidence without relinquishing a single step.

Adjunct means should not be neglected, and bitters, as well as steel medicine, combined with sufficient alimentation, may render good service. As to exercise and gymnastics, which are commonly recommended, they have the inconvenience of occasioning a great expenditure of strength, which the daily alimentation is unable to withstand; these should therefore be reserved until convalescence is well established, and should be used with great caution.

When, by the aid of these precautions, the amount of nourishment has been raised to proper proportions, the patients will be seen to undergo a great change, their strength and condition to return, and their intellectual state to be modified in a most striking manner. It will be prudent, however, for a long time to exercise rigorous watchfulness, and to combat energetically the least retrograde tendency, should such appear. Relapses are in these cases easy; and besides, this form of hypochondria is the index of a nervous predisposition which cannot be noticed without a feeling of uneasiness as to the intellectual future of the subject.

Without wishing to generalize too much on the influence ultimately exercised by the intellectual condition with reference to insufficient alimentation, I think that this is an element which it is well to bear in mind when dealing with many nervous disorders: the majority of hysterical and nervous sufferers make themselves remarkable for the slenderness of their diet, by their liking for indigestible food, and their antipathy for bread, meat, and strengthening dishes. These dispositions are met without any stomachic-nervous disorder, properly so called, for a sustained effort of the will suffices to lead the alimentation back to regular conditions: let this point of practice, then, be insisted on, for the sickly predominance of the nervous system is kept up by the impoverishment of the blood which results from imperfect nutrition; and so long as the patients will not apply their will to nourish themselves in a suitable manner, it will be impossible to reckon upon a solid cure and safety against all danger of relapse.—(*Annales Médico-Psychologiques*, Jan. 1860.)

---

### 3. *On Sitomania.* By Dr. William J. Chipley.

Dr. Chipley applies the term *sitomania* to those forms of insanity which are accompanied by an obstinate rejection of food. He uses the term as a matter of convenience, and not as implying the existence of a distinct form of mania. In certain cases the term *sitophobia* would be most correct, as expressive of the intense dread of food experienced.

Sitomaniacs are classed by Dr. Chipley in two groups: the *first*, including those cases who refuse food, actuated as they believe by a divine command or other supernatural direction, or by the impression that it is dangerous to eat, or that it is morally wrong, or that the food offered is poisoned, or that the stomach and bowels are closed up or are wanting; the *second*, including those cases in which the aversion to food is manifestly associated with chylopoetic derangement. In the first class the cases depend upon some peculiar condition of the brain or state of the mind, the digestive organs remaining apparently intact; in the second class the cases depend upon manifest disorder of the digestive organs.

He does not mean to assert that every case can be readily and certainly placed in one or the other of these two classes. This division shares the fate of all other efforts at classifying diseases. It is necessarily imperfect. We meet with cases where the refusal of food is mainly owing to the existence of some illusion or hallucination, but is strengthened by some digestive derangement which lessens the appetite, and in so far weakens the natural inclination for food. On the other hand, hallucinations are not unfrequently attendants on secret lesions of digestion, and they are apt to enforce an abstinence naturally resulting from an entire absence of appetite. The division is practically important, because the proper treatment of the two classes of patients is radically different. It would be folly to force food on one whose stomach is manifestly incapable of elaborating it, and with whom the assimilative functions are perfectly torpid; and it would be an error of equal magnitude to dose one with physic whose digestive apparatus was in a state of integrity, and who endures the torments of hunger because he has heard a voice commanding him to seek martyrdom. In the one case we should increase existing evils, and augment in no small degree the sufferings of the patient; in the other we should but add physical obstacle to the mental difficulty already in our way.

Dr. Chipley continues:—

"Having regard for my own observation, I have no hesitation in stating that by far the most fruitful cause of sitomania is some morbid condition of the brain giving rise to hallucination, and this is in accordance with the observations of most of the writers whose works I have consulted on this subject. Among the most common delusions is the fear of poison. There is no evidence of physical derangement. The tongue, the pulse, the skin, all may indicate a healthy condition of the digestive apparatus, and hunger may be intense, but the belief that his destruction is sought by poison is so profound, that the patient will endure its torment rather than take the food offered to him. His conversation may be rational, he may enter into the discussion of various topics with animation and with every appearance of perfect judgment, and nothing may seem capable of throwing him off his balance until food is presented, when he will suddenly break off the conversation, and either seek to retreat from the food or exhibit the depth of his suspicions by a minute examination of the articles offered to him, subjecting them to the test of the senses in the most careful manner. Breaking the bread, he will scrutinize the fractured portions, or smell it, or touch it to the tongue, confidently expecting to detect proofs of the truth of his suspicions. In certain instances there is some perversion of taste, and in this case the altered flavour of the food is, to the deluded victim, the highest evidence of the foul wrong that is sought to be inflicted upon him. But whether this perversion of taste exists or not, on closing his examination the patient turns away, often resolving to die of hunger rather than of poison. Sitophobia from an apprehension of poison has been more or less apparent in almost all the cases of obstinate rejection of food which have fallen under my observation.

"A certain number fancy that God has commanded them to fast, sometimes for a definite, at others for an indefinite period of time. These are among the most difficult cases to vanquish, and almost always demand a resort to force. As they firmly believe they are obeying the commands of God, they exhibit all the devoted resolution of the martyr; and many of them would submit to be thrust into a fiery furnace rather than appear to be so impious as to seek to countervail the will of Heaven. Religious fanatics are not unfrequently impressed with the notion that it is their duty to imitate the self-denial of our Saviour, and are thus led to attempt a fast of forty days. One who dreads poison may be frightened into compliance, or he may elect to swallow the food offered rather than have the same, or perhaps more deadly mixtures forced upon him; but the fanatic relies upon the support and succour of Deity in the one case, and consoles himself in the other by the reflection that he is not responsible for what he has no power to avoid.

"In other cases the victim imagines that he is commanded to do penance, as in the case mentioned by Morison, of a married man, who becoming connected with a dissolute woman felt the immorality of the act so deeply that he was rendered insane. He obstinately refused food, alleging that God forbade him to eat.

"There are others who allege that they have communication with spirits, good or evil, and that they are enjoined by them not to partake of food. A case of this kind occurred to me. The patient had been greatly excited on the subject of spirit-rapping, and became insane. He obstinately refused food because, he said, the spirits told him that he would thus purify the body, exalt his spiritual nature, and render himself more worthy of free and unrestricted intercourse with the virtuous dead.

"Some patients have obstinately refused to partake of food under the influence of a vague notion that to eat would dishonour themselves, or in some mysterious manner compromise their friends. In these cases the patients will rarely give, or attempt to give a reason for the fear by which they are agitated. They say they know that such is the case, although they may not be able to explain it; and if food is pressed upon them they become greatly agitated, and offer a resistance which might be expected if you were really seeking to dishonour them or to injure some of their best friends. I have a person now under treatment who for some time before he was brought to the hospital obstinately refused all sustenance, because of a conviction that his family were destined to starvation, and that it would be wrong for him to indulge in a gratification that was soon to be denied to his wife and children. He had been unfortunate in some speculation, and had also lost money by endorsing for a friend, though his fortune was but little impaired. Yet he could see no termination to his misfortunes but extreme poverty and the absolute starvation of his family. When at home, seated at table, bountifully supplied with all that could be desired, he would admit that want was not yet upon them, but it would soon overtake those he so devotedly loved, and it was his duty to be the first sufferer, and by abstinence to leave the more for his wife and child, and thus postpone for them the evil day. The gentleman admitted that his appetite for food was good, that he craved it, and would relish it if what he conceived to be a correct principle did not forbid indulgence. Such feelings have not unfrequently led to terrible tragedies; and the safety of the patient and his family alike demanded immediate seclusion, which I did not hesitate to advise.

"Two years ago a patient was confided to my care who had not partaken of food for more than a week, because, as he alleged, his throat was completely closed, and it was impossible to swallow the least morsel. This was the only evidence of insanity; otherwise he conversed with reason, was sensible of and lamented his unfortunate condition; but no persuasion of his family and friends could induce him to make the effort that would have demonstrated the falsity of his opinion, if it did not dissipate his hallucination. When he came into my hands I lost no time, but, having ascertained by a careful exploration that no obstacle to deglutition existed, and that there was nothing in the condition of the digestive organs to forbid food, I took prompt measures to convince him that the channel was not wholly closed. But he yielded this delusion only for another. He declared that he was only mistaken as to the point of obstruction—that it would be worse than folly to eat when the lower bowel was completely closed, and nothing could pass from him. A dose of oil, indicated by the condition of the bowels, drove him from this last refuge. Lingering faintly, and becoming more or less apparent at times, the delusion ultimately disappeared, and the patient returned to his family in good health.

"Others have supposed that life had ended with them, and reasoning correctly from false premises, they refused to eat, as dead people have never been known to indulge in that sort of luxury. Others have rejected all sustenance, because they laboured under the delusion that immortality had been conferred upon them, and that consequently they had no need for the gross food on which poor mortals subsist. Some are deterred from eating by illusions of the senses. Their food seems to bristle with pins or needles, or they fancy that it is mere filth that is tendered them for food, or it may be that they are convinced that an effort is made to induce them to partake of human flesh or of the flesh of their own children. In all these cases the sense of sight is perverted, and the brain is not in a condition to correct the false sensation.

"In many cases food is deliberately and pertinaciously refused with a view to terminate life, together with all the real or fancied ills to which the poor victim is subjected. It is fortunate, however, that this resolution is much more frequently adopted than persevered in; yet some of the most troublesome and protracted cases are of this description. Nothing can be more astonishing than the strength of will and self-control exhibited by some who have thus sought to destroy themselves. The most wonderful feature is that one, who has determined to quit a life of misery voluntarily, should select the most painful and protracted of all modes of committing suicide. Take, for example, certain cases where the subjects have persevered to the consummation of their purposes, and, during the terrible and protracted agony, have coolly noted their sensations from day to day, until the failing strength could no longer wield the pen. I need not say how difficult is the task of bending this iron will, or of bringing such an one to the abandonment of a purpose so firmly fixed. Yet this has been accomplished, as I shall have occasion to say presently, by very simple means.

"There is another description of cases met with by the general practitioner, but which do not ordinarily fall under the observation of the members of our speciality until they have so far progressed as to have ceased to be wholly mental—the digestive organs having become involved, and appearing then to be principally at fault. I allude to those cases in which a morbid desire for notoriety leads to protracted abstinence from food, in spite of the pangs of hunger, until finally all sustenance is refused. I have never witnessed a case of this kind except in females predisposed to hysteria. These cases are remarkable because they are almost peculiar to well-educated, sensible people, belonging to the higher walks of society, and on any other subject would scorn to deceive or prevaricate, and who, in the language of Dr. Seymour, have nothing 'to gain by pity, except that commiseration, attention, and astonishment, which excite and occupy the mind.' This is another phase of that terrible malady, hysteria, which so often incites its high-born and accomplished victims to most curious attempts at imposition on those around them. But this desire to excite the astonishment of the world by abstinence from food is not more wonderful than the numerous instances on record where sand and pebbles have been introduced into the urethra and passed with the urine as products of the bladder; or the cases of inordinate vomiting sustained for long periods of time by swallowing secretly nauseous substances, while the physician was anxiously labouring to arrest the progress of what he supposed to be a grave form of disease. The intense anxiety of a loving father, the deep, indescribable agony of a devoted mother, the pallid cheeks and fast-falling tears of all who surround the couch, have no other effect on these subjects than that of incentives to carry the gross imposition to extreme lengths. Notoriety is the object—the poor gratification of being pitied and talked of as suffering in a manner and to an extent which no other mortal ever endured, is the paltry reward that lures the victim on to ruin and the grave. And where shall we seek a solution of the problem involved in these cases, save in the morbid condition of the brain; and if this is their source, in what light are we to view these perverted actions but as evidences of insanity? I am one of those who believe that the poet availed himself of the licence of his tribe when he wrote:—

'A rose by any other name would smell as sweet.'

I think there is a great deal in a name, and especially where disease is concerned. I am sure I have known persons to die who might have survived if their malady had been correctly named, and I am pretty certain that I have seen some die in the bloom of youth who might have lived to a green old age, if the practitioner had had the discernment to perceive, and the moral courage to pronounce the true name in such cases as are the subjects of this paragraph before it was too late to rescue the infatuated one from the grave. . . . .

"I presume every gentleman present has met with cases among those confided to his care where food was refused for some time, for the obvious purpose of effecting some particular end, or in revenge for some fancied wrong. With such subjects there exists neither hallucination, illusion, perversion of taste, nor derangement of the digestive organs. They are deliberate attempts to extort some privilege or favour which it may not be thought proper to grant at the time, or mere petty efforts to annoy those having them in charge. The device may have been suggested to the patient by witnessing the anxiety of the physician, in regard to some real case of sitomania, in which he was evidently ready to allow any privilege or favour that promised to effect a compliance with his wishes. For this, among other reasons, the observation of other patients should be always avoided when it becomes necessary to resort to forced alimentation."—(*American Journal of Insanity*, July, 1859.)

---

### 4. *On the Criminal Responsibility of Hysterical Individuals.* By Dr. LEGRAND DU SAULLE.

VERY recently the question has been raised in France of the criminal responsibility of persons subject to attacks of hysteria. A girl had been guilty of child-stealing, in order to impose upon a former lover the belief that she had been pregnant by him. In her defence it was pleaded that her moral liberty had been weakened from her being a subject of hysteria, and on this ground she was acquitted. Dr. Legrand du Saulle discusses and disputes the legitimacy of this decision. He asks, Does hysteria fetter the moral liberty? Does an affection which has its source in a particular sensibility of the nervous system, and not in mental disease, exclude culpability, and transform a crime into a simple fault (*délit*)? To these questions he answers as follows:—

"It is evident that hysteria may well shake a little the edifice of our faculties, properly so called; but in order that no one may consider this an equivocal expression, we ought in the first place to define what we understand by faculties, and to show what is the order of faculties, the exercise of which is liable to be disturbed by the malady in question. Well, then, looking at man from the physiological and psychical points of view, we see that he is the subject of two orders of faculties—the *affective* faculties and the *intellectual* faculties. To the affective faculties belong the phenomena which express a love, a propensity, for certain things, and a hatred, a repulsion, for certain others. To surrender oneself to the affective faculties, being otherwise of sound mind, is to defer to the impulses of the passions; it is to subordinate the actions of life willingly and knowingly to the satisfaction of the desires.

"To the intellectual faculties appertains the gift of enlightening the determinations of the will, and making apparent the conformity or disparity of our actions with the precepts of morality. By the aid of judgment, based on observation and experience, they discover also the consequences of each action.

"From a consideration of the phenomena of hysteria, it may be concluded that this affection might forcibly re-act upon the affective faculties, and in the end might conduce to their injury, but that the intellectual faculties would ordinarily remain intact, the reason assisting in the ruin of the heart, but surviving it.

"The first degree of affective disorder results from the *passions*, the second from insanity. The passions alone being in question in the consideration of hysteria, and the *affectivity* being only obliterated in the first degree by this malady, we need not occupy ourselves with insanity, to which hysteria only leads in prodigiously rare exceptions.

"But if the passions leave to the law full liberty of action in the matter of repression, it is not the less true that they are a very frequent cause of extenuated responsibility, and in certain cases familiar to all, of absolute exoneration from all penalty—as, for example, in the case of the murder of a wife found in flagrant adultery in the conjugal dwelling; or again, where it concerns the crime of castration immediately provoked by a violent outrage upon modesty.

"As no one could promise for himself that at any given moment he would have power to master one of those impetuous motions of the mind under the instantaneous influence of which an action is committed, justice, before applying the rigour of the law, is accustomed to inquire whether at the moment of action there was not a partial eclipse of reason, and if such be the case, she allows the accused the benefit of extenuating circumstances. The culpability is lessened, and the punishment also.

"According to the intensity of the hysteria, and the more or less marked perversion, concomitant or consecutive, of the affective faculties, there ought, we think, to be responsibility or extenuated responsibility, but never, or almost never, should total irresponsibility be allowed for this cause."

From these considerations it follows:—That in hysteria the affective faculties are disordered in various degrees, but the intellect almost always remains intact. That an hysterical condition of weak or even medium intensity, interfering in no way with the perception of the quality of actions committed, it ought not to constitute a title to the indulgence of a tribunal. That hysteria, raised to a high pitch of intensity, carries with it extenuation of responsibility, and consequently of penalty.—(*Annales Médico-Psychologiques*, Jan. 1860.)

---

### 5. *On the Mental State in Chorea.* By Dr. Marcé.

The moral and intellectual faculties are very commonly disordered in cases of chorea. In a given number of instances, two-thirds at least will manifest, in a more or less prominent manner, indications of this psychical disturbance. As to the immunity which is observed in the remaining third, it cannot be explained either by the age or the sex of the subjects, or by the extent or intensity of the convulsive movements.

Four morbid elements, sometimes isolated, more often associated one with another, ought to be studied in the mental state of patients suffering from chorea:—

(1) Disorders of the moral sensibility, consisting in a notable change of the character, which becomes bizarre and irritable; in an unaccustomed tendency to gaiety or to sadness, especially the latter.

(2) Disorder of the understanding, characterized by weakness of memory, and great mobility in the ideas and impossibility of fixing the attention.

(3) Hallucinations, phenomena which until now have never been noted in chorea. These hallucinations supervene in the evening, in the state intermediate between sleeping and waking, more rarely in the morning when awaking, and sometimes whilst dreaming. Often they are limited to the sense of sight, but in rare cases they affect the general sensibility and even the sense of hearing. They are met with in purely uncomplicated chorea, but their occurrence is much more frequent whenever the affection is associated with hysterical symptoms. If, in the majority of cases, these hallucinations constitute a symptom without gravity, they may, under certain exceptional circumstances, induce excitement and delirium.

(4) Finally, chorea may, at its commencement, or during its course, be complicated with maniacal delirium. This gives rise to a very grave state, which in more than half the cases has a fatal termination in the midst of formidable ataxic accidents; and even in the favourable cases, it often induces sundry disordered states of the intellect of variable duration. Inhalations of chloroform, prolonged baths, and antispasmodics are the therapeutic means which have proved most serviceable in the treatment of this delirium, which, in the majority of cases, is to be regarded as a purely nervous affection.—(*Annales Médico-Psychologiques*, Jan. 1860.)

---

### 6. *On Consecutive Cerebral Ramollissement.* By Dr. Adolphe Gubler.

Dr. A. Waller has shown that if the anterior root of a spinal nerve be divided, the nerve tubes towards the peripheral extremity of the nerve quickly become modified in structure, whilst the portion of the nerve-trunk remaining attached to the spinal column retains its normal character. But if the posterior root of the nerve be divided, between the ganglion and the cord, it is, on the contrary, the central portion of the nerve which undergoes a change of structure, whilst the peripheral remains unaltered. Dr. Gubler thinks that this difference in results may be explained by the inverse nature of the two nerve currents, centrifugal in the first case, centripetal in the second: the permanence of the current, that is to say of the function, maintaining integrity of structure, and the cessation of the current or function inducing quickly alteration of structure in the diseased organ. Thus he conceives that we see constantly verified a general law of physiology, to wit, that the organ is made for the function. Whatever the explanation may be, the experimental facts demonstrated by Dr. Waller exist, and Dr. Gubler believes that traumatic or spontaneous lesions, which occasion an organic breach of continuity in the nerves and nervous centres, will bring about structural results and consequences, similar to those observed on experimental division of nerve-cords.

In illustration he relates a fatal case of illness marked by symptoms of cerebral ramollissement, unilateral hemiplegia, with muscular rigidity, and abolition of speech. The autopsy showed plastic infiltration with inflammatory softening of a great portion of the medullary substance of the left hemisphere; and softening of different parts of this hemisphere situated between the first lesion and the spinal cord, and particularly of the inferior portion of the left cerebral peduncle. The pathological changes were, he conceives, to be assigned to two orders of facts, the one active, the other purely passive. The plastic infiltration of the left hemisphere was evidently of an inflammatory character, and had doubtless marked the outset of the affection, giving rise to symptoms of softening with irritation which had been observed four months before the fatal termination. On the contrary, the softening of those portions of the encephalon situated between the centre of the left hemisphere, the seat of the inflammatory change, and the periphery of the body, Dr. Gubler regards as a phenomenon comparable to that alteration of the peripheral portion of a nerve, which follows a section of its anterior root in the vicinity of the cord. If we note the seat of the peduncular softening, the inferior portion of the peduncle, we find that it is precisely this locality in which, according to all anatomists, are found the prolongations of the anterior pyramids, in other terms, of the motor bundles which are about to be distributed to the members. This would follow from what we have premised, these lesions being regarded as peripheral ones in relation to the primitive centre of the cerebral affection. Another circumstance to be noted was, that the softened parts below the region of the hemisphere which was inflamed chronically and infiltrated with plasma, did not present any signs of exaggerated vascular action, any exudation, and nothing which indicated active morbid action: there was seen simply a breaking down of structure, and an accumulation of fatty globules, coming no doubt from the *axis cylinder* of nerve tubes in process of destruction. The softened and nearly deliquescent bed of the left cerebral peduncle seemed to be about to undergo a melting like the putrefaction of a dead fœtus in the uterus, or of a sphacelated organ. Dr. Gubler concludes, therefore, that there was in this case a *primitive lesion* due to active changes of an inflammatory nature, and a *consecutive* and *passive lesion*, depending upon the interruption of the nervous efflux in the bundles of motor nerves. The same thing, he thinks, may have place in all cases of cerebral affections, and it is important to bear this probability in mind.

Lallemand relates (Letter II., obs. 3, § 4) a very extraordinary fact, which may be looked upon as an example of secondary lesion, ascendant or centripetal. A soldier suffered from a traumatic aneurism of the right axillary artery. The vessel was tied, but unfortunately the the brachial plexus was included in the ligatures. The operation was immediately succeeded by excruciating pain in the neck, which often recurred during the following days. To this pain were subsequently joined cerebral phenomena, convulsions, and sinking. Death occurred, and at the autopsy the posterior extremity of the left hemisphere was found softened and greenish, these changes extending to the corresponding lateral ventricle. The softening had proceeded even to diffluence, and in the centre there was more than a spoonful of a thick greenish liquid, which Lallemand considered to be pus.

Dr. Gubler asks, by what mechanism this profound alteration of the left cerebral hemisphere was produced, which arose from and depended upon the nerves of the right brachial plexus? Was it caused by a transmission of irritation, a propagation of inflammation, or a suppression of function? If it were certain that the ultimate cerebral changes were purulent, it would be requisite to have recourse to the first of these hypotheses, and the probability would be in favour of the transmission of irritation, with the creation at the spot of an inflammatory change determined by nervous excitation; but the greenish tint of a ramollissement does not necessarily imply the presence of an infiltrated purulent liquid, certain cerebral gangrenes independent of all inflammatory action having shown a like tint. A doubt then is permissible, and the idea of the case being one of *ramollissement atrophique* is not improbable. Dr. Gubler thinks, also, that other cases are on record which support his views. For example, M. Charcot has published a case of atrophy of a cerebral hemisphere, coinciding with atrophy of the spinal cord on the opposite side; and M. Luys has communicated to the Society of Biology the result of his researches upon a case of alteration of certain nerves of the members, as the sequel of an attack of hemiplegia of cerebral origin.

Dr. Gubler terminates his observations by the following conclusions:—

1. It is necessary to distinguish, in affections of the nervous system, two classes of lesions: the one promordial and essentially variable; the other secondary, or consecutive.

2. The consecutive alterations are sometimes localized around the protopathic lesions, sometimes transmitted to a distance. The first, long known, are occasioned by eliminating or irritating inflammation, and consist in circumferential softenings, ventricular or sub-arachnoid effusions, resorption of tissues, formations of cysts, &c.

3 The secondary lesions, propagated to a distance, and newly submitted to observation, appear to be of two kinds—active and passive.

4. Those resembling the retrograde transformations undergone by tumours which have ceased their evolutions, or by a fœtus which has died in the uterus, ought to be considered as the result of an abolished or enfeebled nutrition; in a word, of atrophy. And as these changes are characterized by a diminution of cohesion of the nervous substance, extending even to diffluence, it will be convenient to apply to them the denomination *atrophic softening—ramollissement atrophique.*

5. This atrophy appears to be linked to the suppression of the functions of the part which is the seat of it; consequently, a protopathic lesion being given, there will be secondary passive ramollissement in two directions; on the one hand, between the primitive lesion and the central parts, affecting the bundles devoted to feeling; on the other, between the same lesion and the periphery, affecting the conductors of movement.

6. Thus the softened tracks in the one and the other direction, studied by attentive observers, will serve to fix the respective situation and position of the sensitive and motor fibres in the cords, as well as in the nervous centres. Here still pathology will furnish light to anatomy and physiology.

7. Clinical observation has not yet given us any information upon the particular symptoms of secondary atrophic ramollissements; but we can foresee that in their progress the phenomena of excitement, such as muscular rigidity, will cease, provided that the long duration of the primitive affection has not given place, in the muscles, to changes of condition which are opposed to the mobility of the parts.—(*Archives Générales de Médecine*, July, 1859.)

---

### 7. *On Interdiction.* By M. H. De Castelnau.

M. Castelnau examines the question of interdiction physiologically and legally, and arrives at conclusions differing very considerably from those usually entertained on the subject. He considers that the law of interdiction, although ostensibly instituted for the benefit of the lunatic and his family, sins egregiously against both the one and the other, and that it should be removed from civilized legislation. This law he holds is the means of effecting much injury to the civil and personal rights and the property of many individuals—injury which is in no degree compensated by the therapeutical good which is supposed to be, but which in reality rarely is, obtained from having recourse to interdiction. We shall not follow M. Castelnau in his arguments, but solely confine ourselves to one or two points which may serve to indicate his method of thinking.

M. Castelnau thinks that interdiction is rather unfavourable than favourable to the interests of a family, these being rightly understood.

"The bonds," he says, "which unite all its members constitute the essence even of a family; the more these bonds are straitened, the more solid is the foundation which a family gives to social order. All law ought, therefore, to have for object to draw tighter these bonds in a measure compatible with liberty; and interdiction, so far from cementing these bonds, is in effect a powerful solvent. Before being pronounced, it places the family in opposition, if it be in enmity with the pretended lunatic, and we can certainly predict that, if the least resistance be exhibited on his part to the first steps, all feeling of affection, or even of mutual kindness between him and his family, is irrecoverably destroyed.

"The first effect of the measure is to place those who demand interdiction in one of those situations which the immortal melancholic of Geneva said that we should apply ourselves to avoid; which, indeed, place our duties in opposition to our interests, and which show us our welfare in the ills of others. 'In such situations,' he said, 'whatever be our virtue, it will decline sooner or later without our being aware of it, and we shall become unjust and wicked in act, without having ceased to be just and good in soul.' Observation shows that in the case under consideration, this happens too often."

M. Castelnau conceives also that the law in prohibiting the marriage of lunatics has exceeded its just bounds. He thinks, moreover, that—

"Medicine has equally lost sight of important truths of physiology, to wit, that the natural appetites never lose their rights, and that is that which concerns the sexual functions; monogamous marriage is not solely the best social condition to accomplish them, but also the most salutary. To interdict, therefore, marriage to all insane, is then to neglect the rules of hygiène, to injure the interests of the family that they wish to serve, and to pay a legal tribute to immorality."

M. Castelnau terminates his remarks by several considerations upon the rights and interests of society in their relations with the rights and interests of lunatics.

"The rights of society upon the individual may be reviewed in a word. Every citizen has a right to live free, who does not interfere with the liberty of another. He who cannot effect his good without compromising the liberty and security of his fellows, it is evident that society ought to have the right of taking against him all necessary measures to shield itself; but these measures have not and ought not to have anything in common with interdiction. Moreover, society cannot punish on suspicion of a presumed danger; it is necessary that the danger be manifest. Science often assumes the pretext of fore-seeing, and sometimes justifies this, sometimes not; to authorize preventive measures, it is requisite that science should never deceive itself; still it is probable that many excellent persons recoil before the idea of inflicting a punishment for a fault which is not yet committed.

"This day the law suffers to wander freely in our cities relapsed criminals; it is demonstrated by experience that the majority of them, if not all, renew their criminal attempts against the person or against property, but the law respects their liberty until the committal of the act which can be most surely foreseen. How then can society be more rigorous with those who are thought to have lost their reason, than with those that they regard as acting in the plenitude of their freewill? Not only is it repugnant to the notion o. equity to punish for presupposed facts, but true justice, that is to say, enlightened justice, would have it that society used its rights with moderation for accomplished facts, and that it should not show itself eager to rank among punishable actions perfectly innocent extravagances, as too often happens *vis-à-vis* of lunatics."

M. Castelnau's opinions will at least rank among the curiosities of psychological literature.—(*Brown-Séquard's Journal de Physiologie*, October, 1859.)

